# Chronic Endocannabinoid System Stimulation Induces Muscle Macrophage and Lipid Accumulation in Type 2 Diabetic Mice Independently of Metabolic Endotoxaemia

**DOI:** 10.1371/journal.pone.0055963

**Published:** 2013-02-05

**Authors:** Lucie Geurts, Giulio G. Muccioli, Nathalie M. Delzenne, Patrice D. Cani

**Affiliations:** 1 Université catholique de Louvain, Louvain Drug Research Institute, Metabolism and Nutrition Research Group, Brussels, Belgium; 2 Bioanalysis and Pharmacology of Bioactive Lipids Laboratory, Louvain Drug Research Institute, Université catholique de Louvain, Brussels, Belgium; Paris Institute of Technology for Life, Food and Environmental Sciences, France

## Abstract

**Aims:**

Obesity and type 2 diabetes are characterised by low-grade inflammation, metabolic endotoxaemia (i.e., increased plasma lipopolysaccharides [LPS] levels) and altered endocannabinoid (eCB)-system tone. The aim of this study was to decipher the specific role of eCB-system stimulation or metabolic endotoxaemia in the onset of glucose intolerance, metabolic inflammation and altered lipid metabolism.

**Methods:**

Mice were treated with either a cannabinoid (CB) receptor agonist (HU210) or low-dose LPS using subcutaneous mini-pumps for 6 weeks. After 3 weeks of the treatment under control (CT) diet, one-half of each group of mice were challenged with a high fat (HF) diet for the following 3-week period.

**Results:**

Under basal conditions (control diet), chronic CB receptor agonist treatment (i.e., 6 weeks) induced glucose intolerance, stimulated metabolic endotoxaemia, and increased macrophage infiltration (*CD11c* and *F4/80* expression) in the muscles; this phenomenon was associated with an altered lipid metabolism (increased *PGC-1α* expression and decreased *CPT-1b* expression) in this tissue. Chronic LPS treatment tended to increase the body weight and fat mass, with minor effects on the other metabolic parameters. Challenging mice with an HF diet following pre-treatment with the CB agonist exacerbated the HF diet-induced glucose intolerance, the muscle macrophage infiltration and the muscle's lipid content without affecting the body weight or the fat mass.

**Conclusion:**

Chronic CB receptor stimulation under basal conditions induces glucose intolerance, stimulates metabolic inflammation and alters lipid metabolism in the muscles. These effects worsen following the concomitant ingestion of an HF diet. Here, we highlight the central roles played by the eCB system and LPS in the pathophysiology of several hallmarks of obesity and type 2 diabetes.

## Introduction

Obesity and associated metabolic disorders, such as type 2 diabetes and insulin resistance, are related to low-grade inflammation [1], which may be due to an increase in plasma lipopolysaccharide (LPS) levels, a condition that is defined as metabolic endotoxaemia [2]. Obesity is also characterised by increased endocannabinoid (eCB)-system tone [3], [4]. The eCB-system is composed of endogenous bioactive lipids that activate specific G-coupled receptors, namely receptor CB1 and receptor CB2. Among these lipids, the best characterised are anandamide (AEA) and 2-arachidonoylglycerol (2-AG) [5]. Dysregulation of eCB levels may contribute to pathological conditions such as obesity and type 2 diabetes [5]–[7]; hence, several clinical trials have assessed the effects of CB1 antagonists on weight loss and the improvement of lipid and carbohydrate metabolism [8]–[12]. These effects are only partially explained by reductions in food intake and energy intake, strongly suggesting that the eCB-system has other metabolic effects [13], [14]. We and others have found that blocking the CB1 receptor abolishes the low-grade inflammation that is associated with obesity [6], [15]. Moreover, LPS is known to stimulate eCB synthesis [6], [16], and we recently demonstrated metabolic interactions between LPS and the eCB system in obesity [6], [17]. Indeed, we have shown that LPS produced by the gut microbiota plays a crucial role in the onset of adipose tissue inflammation [2], [18]–[20] and have proposed that the eCB system might be the link between adipose tissue metabolism and gut microbiota [6]. Various studies have revealed the involvement of the eCB system in the modulation of glucose and energy homeostasis in humans and in animal models of obesity [21]–[26]. More recently, it has been demonstrated that the acute stimulation of CB1 influences insulin sensitivity, an effect that is apparently mediated by the CB1 receptor in skeletal muscle [27]. The effects of the eCB system on glucose homeostasis and muscle metabolism thus appear to be a putative mechanism that explains the metabolic alterations associated with obesity and type 2 diabetes. To date, it remains to be demonstrated *in vivo* by which mechanisms the chronic stimulation of cannabinoid receptors or the mimicking of metabolic endotoxaemia affects muscle metabolism and glucose homeostasis. Therefore, to address this question, we chronically treated mice with either a cannabinoid (CB) receptor agonist (HU210) or low-dose LPS prior to and during a high fat (HF) diet challenge.

## Materials and Methods

### Mice

A set of 9-week-old male C57BL/6J mice (46 mice, n = 7–9/group) (Charles River Laboratories International Inc., Brussels, Belgium) were housed in a controlled environment (12-h daylight cycle, lights-off at 6 pm, controlled temperature and humidity) in groups of 2 mice per cage, with free access to food and water. After one week of acclimatisation, all mice were put on a control diet (CT) (A04, Villemoisson sur Orge, France). After 3 weeks of being fed the CT diet, one-half of the mice were randomly assigned to 3 groups receiving a HF diet (HF) (D12492, Research diet, New Brunswick, NJ, USA) containing 60% fat (kcal/100 g) and 20% carbohydrates (kcal/100 g) for an additional 3 weeks. The remaining mice received treatments while on the CT diet (Figure S1). The weight of each mouse was monitored twice a week. This study was performed in strict accordance with the guidelines of the local ethics committee (the ethical committee of the Université catholique de Louvain for animal experiments specifically approved this study, which received the agreement n° 2010/UCL/MD/022) and in accordance with European recommendation 2007/526/CE, which was transformed into the Belgian Law of April 6, 2010, regarding the protection of laboratory animals. Our laboratory received agreement number LA1230314 from the Ministry of Agriculture. All animals were housed in cages that provided an environment with controlled light/dark cycles, temperature and humidity. Every effort was made to minimise suffering during the manipulations and oral gavage. Food was removed 2 h after the onset of the daylight cycle, the mice were fasted for 6 h, and all animals were then anaesthetised with ketamine-xylazine before exsanguination and tissue sampling. Subsequently, the mice were killed by cervical dislocation.

### Surgical procedures for the implantation of osmotic mini-pumps and *in vivo* treatments

At treatment day 0, the mice were anaesthetised with isoflurane (Forene®, Abbott, Queenborough, Kent, England) and implanted subcutaneously with a mini-osmotic pump (Alzet Model 2006, ALZA, USA) (flow rate: 0.15 µl/h, total filling volume: 200 µl, delivery duration: 42 days). Using these mini-osmotic pumps, the mice were infused with HU210 (50 µg/kg/day) (Tocris). HU210 is a well-known and potent agonist [28] of the first identified receptors CB1 and CB2 and, to a lower extent, other endocannabinoid-related receptors (i.e., GPR55, Peroxysome Proliferator Activated Receptors alpha/gamma) [23], [29], [30]; moreover, the drug has already been demonstrated to exert effects on metabolism *in vitro* [23] and *in vivo* [6]. In preliminary experiments (not shown), we selected a dose that permitted chronic treatment with the cannabinoid agonist without central adverse effects (i.e., reduced locomotion and a cataleptic state) while maintaining the peripheral effects of the treatment; we thus ensured an accurate investigation of the chronic CB receptor agonist during the six weeks. A second group of mice received LPS from *Escherichia coli* 055:B5 at a concentration of 300 µg/kg/day (Sigma), and a third group received vehicle (0.1% Tween/saline) for the entire 6-week period.

### Oral glucose tolerance test

The oral glucose tolerance test was performed after 5 weeks of treatment (i.e., 2 weeks of HF-diet treatment). The food was removed two hours after the onset of the daylight cycle, and the mice were treated after a 6-h fast, as previously described [2], [31]. Twenty microliters of plasma were collected 30 min before and 15 min after the glucose load to determine the plasma insulin concentrations.

### Tissue sampling

The mice were anaesthetised by an intraperitoneal injection of 100 mg/kg ketamine and 10 mg/kg xylazine after a 6-h fast. Portal-vein blood samples were harvested for further analysis. Subsequently, the mice were killed by cervical dislocation. White adipose tissue deposits (subcutaneous: inguinal and lower back deposits), the *tibialis anterior* (TA) skeletal muscles, and the cerebellum were precisely dissected, weighed and immediately immersed in liquid nitrogen and stored at -80 °C for further analysis.

### Biochemical analyses

The plasma insulin concentrations were determined using an ELISA kit (Mercodia, Uppsala, Sweden) in 20 µl of plasma collected from tail blood during OGTT and measured according to the manufacturer's instructions.

The portal plasma LPS concentration was measured using the Endosafe-MCS detection system (Charles River Laboratories, Lyon, France), based on the Limulus amoebocyte lysate (LAL) kinetic chromogenic methodology, as previously described [32].

The plasma non-esterified fatty acid (NEFA) concentrations were measured via kits utilising an enzymatic reaction and the spectrophotometric detection of the reaction end products (Diasys Diagnostic and Systems, Holzheim, Germany), according to the manufacturer's instructions.

### Muscle histological analysis

For the detection of neutral lipids, frozen sections obtained from a fraction of the *tibialis anterior* muscle were sliced and stained with Oil Red O, using 0.5% Oil Red O dissolved in propylene glycol for 10 min at 60 °C. The slide sections were then counterstained, and twenty fields per animal were analysed. These measurements were performed by an investigator who was blinded to the experimental groups.

### **RNA preparation and real-time qPCR analysis**

Total RNA was prepared from the tissues using the TriPure reagent (Roche Diagnostics Belgium, Vilvoorde). Quantification and integrity analysis of the total RNA were performed by running 1 µl of each sample on an Agilent 2100 Bioanalyzer (Agilent RNA 6000 Nano Kit, Agilent). Subsequently, cDNA was prepared by reverse transcription of 1 µg of total RNA using a Reverse Transcription System kit (Promega, Leiden, The Netherlands). Real-time PCR was performed with the StepOnePlus™ real-time PCR system and software (Applied Biosystems, Nieuwerkerk aan den Ijssel, The Netherlands) using Mesa Fast qPCR™ (Eurogentec, Seraing, Belgium) for detection, according to the manufacturer's instructions. RPL19 RNA was selected as the housekeeping gene. The primer sequences are presented in Table 1. All samples were run in duplicate in a single 96-well reaction plate, and the data were analysed according to the 2^−ΔΔCT^ method. The identity and purity of the amplified products were assessed by analysing the melting curves that were obtained after completion of the amplification.

**Table 1:** Primers sequences.

Primers	Forward Sequence	Reverse Sequence
RPL-19	GAAGGTCAAAGGGAATGTGTTCA	CCT GT TG CT CA CTTGT
CB1	CTGATGTTCTGGATCGGAGTC	TCTGAGGTGTGAATGATGATGC
CB2	CCCTACCTGTAATCCCAGCA	TTGAGGTAAGGGGGTCTCAA
F4/80	TGACAACCAGACGGCTTGTG	GCAGGCGAGGAAAAGATAGTGT
CD11c	ACGTCAGTACAAGGAGATGTTGGA	ATCCTATTGCAGAATGCTTCTTTACC
GLUT-4	GGGAAGGAAAAGGGCTATGCTG	CAATGAGGAACCGTCCAAGAATG
LPL	TCTGTACGGCACAGTGG	CCTCTCGATGACGAAGC
CPT-1b	AGCACACCAGGCAGTAGCTT	AGGATGCCATTCTTGATTCG
PGC-1α	AGCCGTGACCACTGACAACGAG	GCTGCATGGTTCTGAGTGCTAAG
CD36	GCCAAGCTATTGCGACATGA	ATCTCAATGTCCGAGACTTTTCAAC
PPARγ	CTGCTCAAGTATGGTGTCCATGA	TGAGATGAGGACTCCATCTTTATTCA
GPR55	ATTTGGAGCAGAGGCACGAACATGA	AGTGGCGATATAGTCCAGCTTCCT

**RPL-19**: ribosomal protein L19; **CB1**: Cannabinoid Receptor 1; **CB2**: Cannabinoid Receptor 2; **F4/80**: macrophage marker F4/80; **CD11c**: cluster of differentiation 11c; **GLUT-4**: Glucose Transporter 4; **LPL**: Lipoprotein Lipase; **CPT-1b**: Carnitine Palmitoyl Transferase 1B; **PGC-1α**: Peroxisome proliferator-activated receptor gamma coactivator -1 alpha, **CD36**: cluster of differentiation 36/Fatty Acid translocase (FAT), **GPR55**: G Protein Coupled Receptor 55.

### HU210 detection and endocannabinoid quantification by HPLC-MS

Mouse cerebella were homogenised in CHCl_3_ (10 ml) in the presence of internal standards (CP-55,940 for HU210; d_5_-2-AG and d_4_-AEA for the endocannabinoids). Subsequently, MeOH (5 mL) was added, and the suspension was sonicated in an ice bath for 5 min. Following the addition of H_2_O (5 mL), the mixtures were vigorously mixed to extract the analytes in the organic phase. The phase separation was completed by centrifuging the mixtures for 10 min at 2500 rpm, and the organic layer was recovered and dried under nitrogen and mild heating. The residue was solubilised in CHCl_3_ and pre-purified by solid-phase extraction using a silica column. Following the CHCl_3_ elution, HU 210 and the endocannabinoids (as well as their respective internal standards) were recovered using a 1:1 mixture of ethylacetate-acetone [33], [34]. The resulting fraction was analysed by HPLC-MS using a LTQ Orbitrap mass spectrometer (ThermoFisher Scientific) coupled to an Accela HPLC system (ThermoFisher Scientific). Analyte separation was achieved using a C-18 Supelguard pre-column and a Supelcosil LC-18 column (3 µm, 4×150 mm) (both from SigmaAldrich). Mobile phases A and B were composed of MeOH-H_2_O-ammonium hydroxide 75:25:0.1 (v/v/v) and MeOH-ammonium hydroxide 100:0.1 (v/v), respectively. The gradient (0.5 ml/min) was designed as follows: from 100% A to 100% B in 15 min, followed by 10 min at 100% B and subsequent re-equilibration at 100% A. MS analysis in the negative mode using the Orbitrap mass analyser was performed using an ESI ionisation source. HU210 was detected (with 2-ppm precision) as a MS^2^ fragment corresponding to the loss of a hydroxyl group (m/z = 367.26420). 2-AG and AEA were detected as their [M-H]^−^ ions and quantified using their respective deuterated standards [35]), followed by normalisation to the tissue weight.

### Validation of the HU210 stability and recovery in tissues after 6 weeks of administration

To confirm the stability of the compound and its accurate *in vivo* delivery after 6 weeks of osmotic-mini-pump usage, we analysed the presence of HU210 by HPLC-HRMS in the brain tissue. The HPLC-MS^2^ trace clearly demonstrated the presence of HU210 in HU and HF-HU treated mice (Figure S2).

### Statistical analyses

The data are expressed as the mean±SEM. Differences between the groups were assessed using one-way ANOVA, followed by Bonferroni's *post hoc* test. A two-way ANOVA analysis on repeated measures was performed for the evolution of glycaemia during the OGTT. The data were analysed using GraphPad Prism version 5.00 for Windows (GraphPad Software, San Diego, CA, USA). The results were considered statistically significant at *P*<0.05.

## Results

### Chronic CB receptor agonist treatment, but not LPS treatment, increases CB1 and CB2 mRNA levels in skeletal muscle but not in adipose tissue

**Figure 1:**
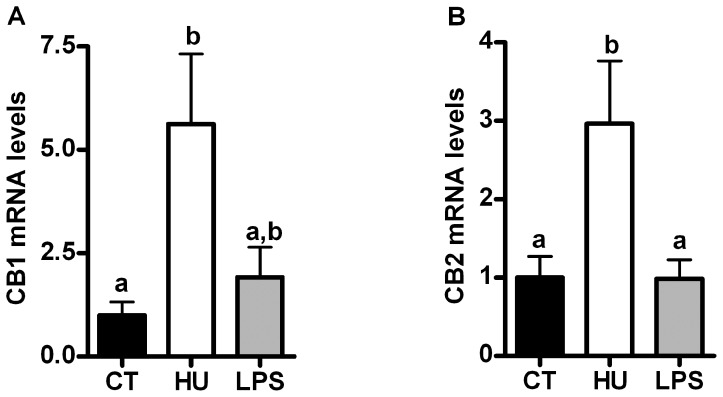
Cannabinoid receptor expression after chronic CB receptor agonist treatment or LPS treatment. (A) *CB1* mRNA levels and (B) *CB2* mRNA levels in the *tibialis anterior* muscle. All results are the means±SEM for 7–9 mice per group from the HU-treated group (HU) and the LPS-treated group (LPS) compared with the results of the control group (CT). The data with different superscripted letters are significantly different based on the one-way ANOVA followed by the Bonferroni *post hoc* test.

Under basal conditions (i.e., a control diet), chronic treatment with the cannabinoid receptor agonist HU210 increased both *CB1* and *CB2* receptor mRNA expression in the skeletal muscle (*tibialis anterior* [TA]), whereas their expression was not affected in the adipose tissue (data not shown). LPS treatment did not affect the CB receptors mRNA levels (Figures 1A and 1B).

### Chronic CB receptor agonist treatment induces glucose intolerance

Chronic infusion of the CB receptor agonist did not affect the food intake, the body weight gain or the weight of the white adipose tissue after 6 weeks of treatment, whereas the LPS treatment increased the body weight gain by 44% and increased the adipose tissue weight compared with the control mice (Table 2).

**Table 2:** SAT weight, body weight gain, food intake and NEFA's levels following treatments.

	CT	HU	LPS	HF	HF-HU	HF-LPS
SAT weight (g)	0,41±0,04^a^	0,37±0,01^a^	0,44±0,01^a^	1,26±0,21^b^	0,99±0,06^b^	1,08±0,11^b^
Body weight gain (g)	2,29±0,27^a^	2,59±0,33^a^	3,03±0,2^a^	6,24±0,99^b^	4,75±0,58^b^	5,67±0,49^b^
Cumulative Food Intake (kcal/mouse/6 weeks)	426,82±14,58^a^	431±13.67^a^	438,06±20,33^a^	530,55±8,48^b^	506,85±28,26^a,b^	484,76±4,19^a,b^
NEFA's (mM)	0.31±0.02^a^	0.41±0.03^a^	0.39±0.04^a^	0.41±0.05^a^	0.45±0.04^a^	0.5±0.04^a^

Subcutaneous adipose tissue (SAT) weight (g); body weight gain (g), cumulative food intake (kcal/mouse/6 weeks) and NEFA's levels in the different treated groups. All results are mean±s.e.m. for 7–9 mice per group. Data with different superscript letters are significantly different according to the one way ANOVA followed to the Bonferroni *post hoc* test.

**Figure 2:**
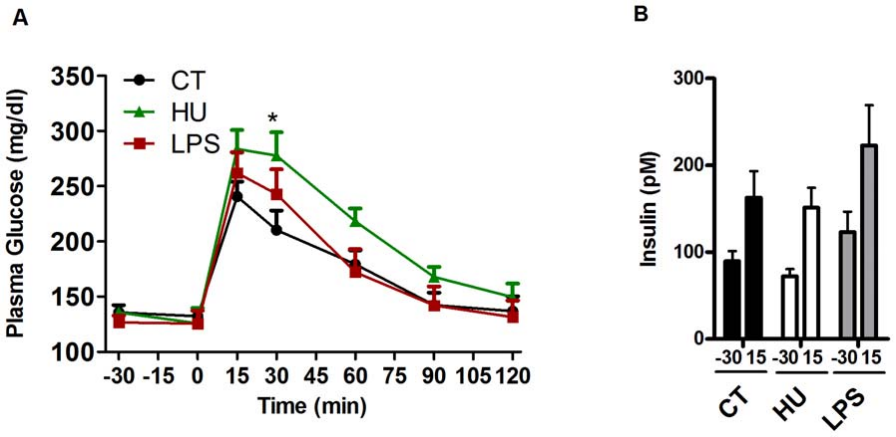
Oral glucose tolerance test and insulin levels after chronic CB receptor agonist treatment or LPS treatment. (A) Plasma glucose profile subsequent to a 2 g/kg glucose oral challenge in freely mobile mice. Two-way ANOVA followed by the Bonferroni *post hoc* analysis revealed the time (p<0.0001), treatment (p = 0.195) and interaction (p = 0.046) effects. * indicates a significant difference (p<0.05) versus CT, as determined by a two-way ANOVA followed by the Bonferroni *post hoc* test. (B) Plasma insulin levels at 30 min before glucose loading and 15 min after glucose loading. All results are expressed as the means±SEM for 7–9 mice per group from the HU-treated group (HU) and the LPS-treated group (LPS) compared with the plasma insulin levels of the control group (CT).

Chronic CB receptor agonist treatment significantly increased glycaemia following an oral glucose load (Figure 2A) at the 30-min time point; furthermore, the glycaemia remained elevated throughout the test, but did not reach significance. The LPS treatment did not significantly affect the glycaemia. The CB receptor agonist did not increase the level of fasting plasma insulin or glucose-induced insulin secretion compared with the control group, whereas the LPS treatment increased the plasma insulin level both before (40%) and after (37%) the oral glucose load, but did not reach significance (Figure 2B).

### Both chronic CB receptor agonist treatment and LPS treatment increase macrophage infiltration and metabolic endotoxaemia

**Figure 3:**
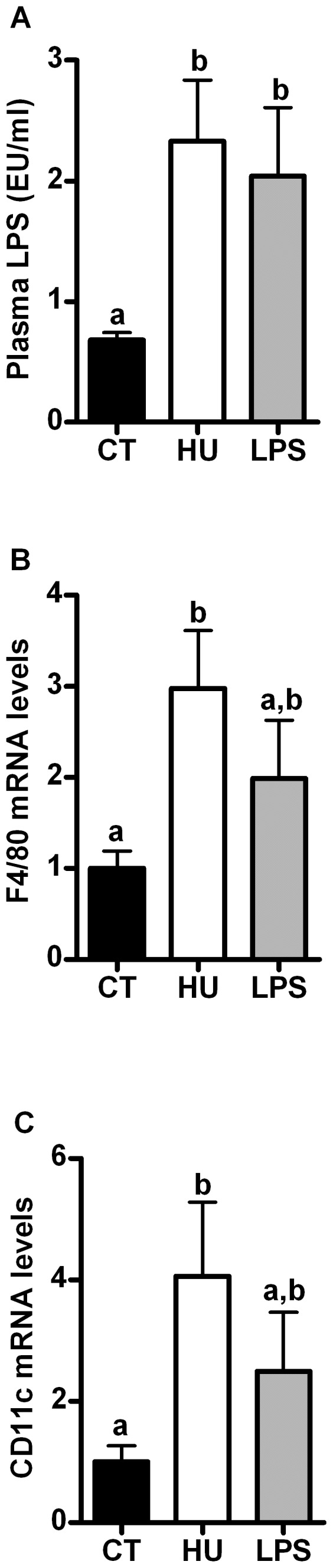
Plasma LPS levels and inflammation markers after chronic CB receptor agonist treatment or LPS treatment. (A) Plasma LPS levels; (B) *F4/80* mRNA levels in the *tibialis anterior* muscle; (C) *CD11c* mRNA levels in the *tibialis anterior* muscle. All results are expressed as the means±SEM for 7–9 mice per group from the HU-treated group (HU) and the LPS-treated group (LPS) compared with the levels in the control group (CT). The data with different superscripted letters are significantly different based on a one-way ANOVA followed by the Bonferroni *post hoc* test.

Both treatments increased the plasma LPS levels (metabolic endotoxaemia) (Figure 3A). The CB receptor agonist significantly elevated the mRNA levels of *F4/80* (3-fold increase) and *CD11c* (4-fold increase) in the TA muscle, whereas the LPS treatment tended to increase these markers (2-fold increase) (Figures 3B and 3C). Chronic LPS treatment significantly increased the mRNA expression levels of the adipose tissue macrophage infiltration marker *F4/80*, whereas the CB receptor agonist increased the *F4/80* mRNA expression by approximately 70% in the adipose tissue, but did not reach significance (Figure S3).

### Chronic CB receptor agonist treatment and LPS treatment differentially affect expression of genes involved in muscle lipid oxidation

To elucidate the metabolic markers that are affected by either CB receptor stimulation or LPS, we investigated the expression of glucose transporter *GLUT-4* and lipoprotein lipase (*LPL*) in the adipose tissue and TA muscle. None of the treatments affected the *GLUT-4* or *LPL* mRNA expression, whether in the TA muscle or adipose tissue (Figure S4).

Plasma levels of non-esterified fatty acids (NEFA) contribute to the development of glucose intolerance. Here, we determined that both pharmacological treatments increased the plasma NEFA by approximately 30% but did not reach significance (Table 2). Interestingly, chronic CB receptor agonist treatment, but not LPS treatment, modified the lipid oxidation in the TA muscle. We found that the CB receptor agonist treatment decreased *CPT-1b* mRNA expression but increased *PGC-1α* mRNA expression (Figures 4A and 4B). The mRNA expression of the fatty acid transporter *CD36* in the TA muscle was not affected by the treatments (Figure S4).

**Figure:**
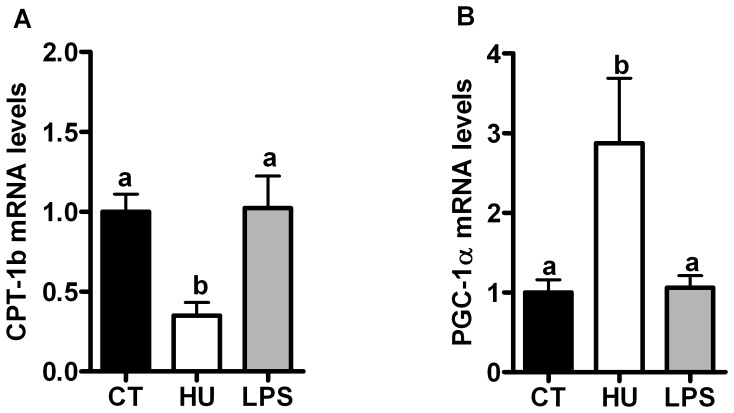
Lipid metabolism marker mRNA levels after chronic CB receptor agonist treatment or LPS treatment. (A) *CPT-1b* mRNA levels; (B) *PGC-1α* mRNA levels in the *tibialis anterior* muscle. All results are expressed as the means±SEM for 7–9 mice per group from the HU-treated group (HU) and the LPS-treated group (LPS) compared with the levels in the control group (CT). The data with different superscripted letters are significantly different based on a one-way ANOVA followed by the Bonferroni *post hoc* test.

### Chronic CB receptor agonist pre-treatment or LPS pre-treatment exacerbate HF diet-induced glucose intolerance

**Figure 5:**
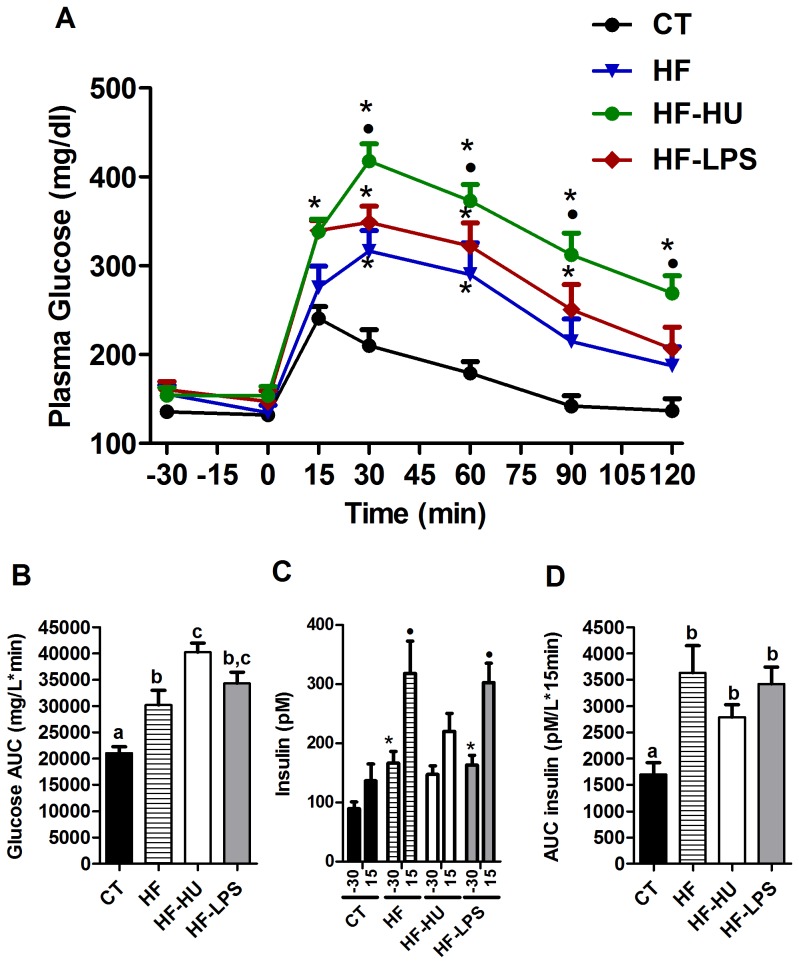
Oral glucose tolerance tests and insulin levels under the HF diet. (A) Plasma glucose profile following an oral challenge with 2 g/kg glucose in freely mobile mice. A two-way ANOVA followed by the Bonferroni *post hoc* analysis revealed the time (p<0.0001), treatment (p<0.0001) and interaction (p<0.0001) effects. * indicates a significant difference (p<0.05) versus CT, and • indicates a significant difference (p<0.05) versus HF as determined by a two-way ANOVA followed by the Bonferroni *post hoc* test. (B) Glucose areas under the curves (AUCs), measured between 0 and 120 min after glucose loading. The data with different superscripted letters are significantly different based on a one-way ANOVA followed by the Bonferroni *post hoc* test. (C) Plasma insulin levels at 30 min before glucose loading and 15 min after glucose loading. * indicates a significant difference (p<0.05) versus the CT time (−30 min), as determined by a one-way ANOVA test. • indicates a significant difference (p<0.05) versus the CT time (15 min) as determined by a one-way ANOVA. (D) Insulin areas under the curves (AUCs) measured between the basal time and 15 minutes after glucose loading. The data with different superscripted letters are significantly different based on a one-way ANOVA followed by the Bonferroni *post hoc* test. All results are expressed as the means±SEM for 7–9 mice per group from the HF diet-treated group (HF), the HF- and HU-treated group (HF-HU) and the HF- and LPS-treated group (HF-LPS) or the control group (CT).

The two pre-treatments did not significantly affect the food intake, the body weight gain or the fat mass (Table 2). However, glucose intolerance was strongly exacerbated after concomitant treatment with an HF diet and the CB receptor agonist (HF-HU group) (Figures 5A and 5B). The LPS pre-treatment (HF-LPS group) significantly increased the plasma glucose level 15 min after the oral glucose challenge, relative to the level in mice treated with the HF diet alone, and the glucose levels remained higher during the subsequent time points (Figure 5A). Importantly, the CB receptor agonist pre-treatment completely abrogated the glucose-induced insulin secretion that was observed after administration of the HF diet, whereas the LPS pre-treatment had no additional effect compared with the HF diet (Figure 5C). However, the mean insulin area under the curve that was measured between the basal time point and 15 min after the glucose load remained significantly higher in the three HF-treated groups compared with the control mice (Figure 5D).

### Chronic CB receptor agonist treatment worsens HF diet-induced inflammation in skeletal muscle

**Figure 6:**
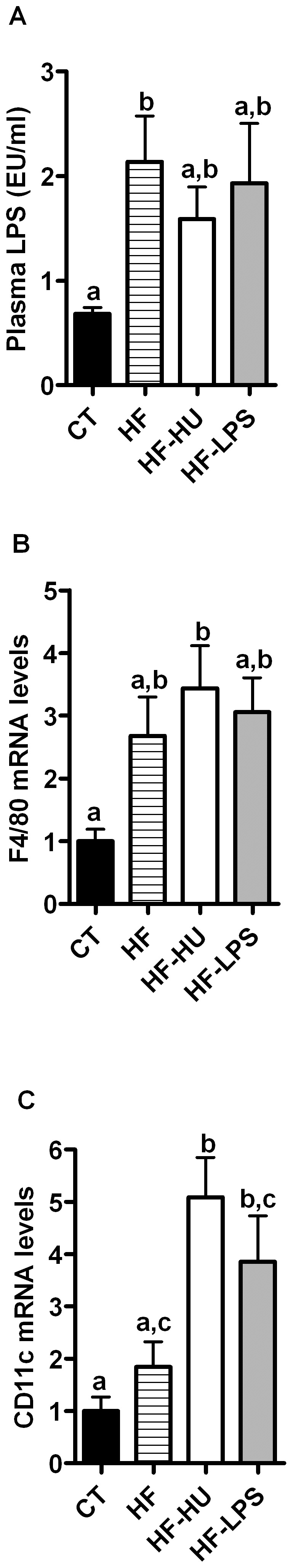
Plasma LPS and inflammation markers under HF diet. (A) Plasma LPS levels; (B) *F4/80* mRNA levels; (C) *CD11c* mRNA levels in the *tibialis anterior* muscle. All results are presented as the means±SEM for 7–9 mice per group from the HF diet-treated group (HF), the HF-and HU-treated group (HF-HU) and the HF-and LPS-treated group (HF-LPS) compared with the levels in the control group (CT). The data with different superscripted letters are significantly different based on a one-way ANOVA followed by the Bonferroni *post hoc* test.

Cannabinoid agonist-treated mice challenged with an HF diet exhibited significantly higher *CD11c* mRNA levels (2.5-fold) than were exhibited after the HF diet alone (Figure 6C), and the serum LPS levels and the *F4/80* mRNA levels were similarly affected by the HF-diet treatment (Figures 6A, 6B and 6C). The LPS-treated mice exhibited similar increases in the serum LPS levels, the *CD11c* mRNA and the *F4/80* mRNA levels compared with the HF-fed mice (Figures 6A, 6B and 6C).

### Chronic CB receptor agonist pre-treatment increases the lipid content in muscles

**Figure 7:**
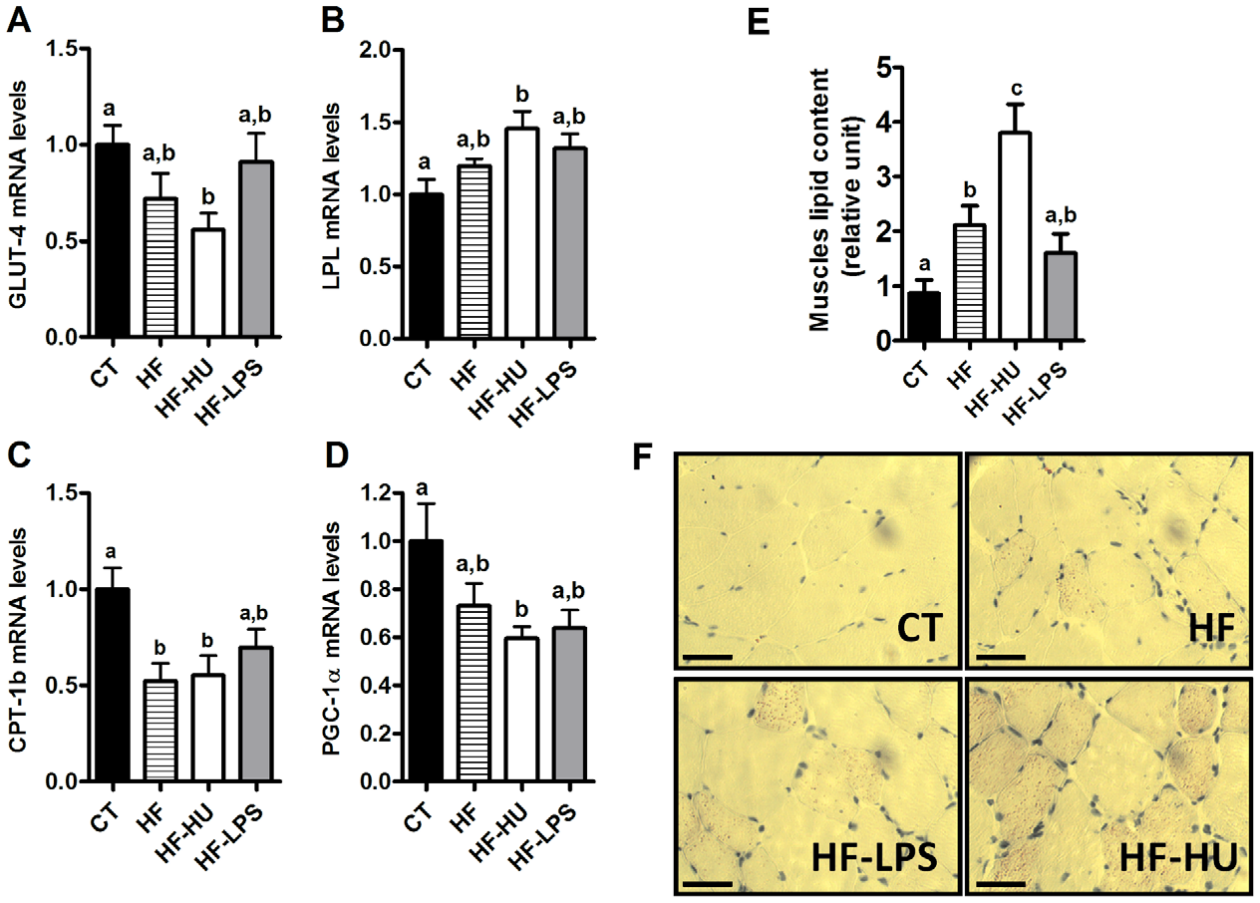
Muscle lipid metabolism markers under the HF diet. (A) *GLUT-4* mRNA levels; (B) *LPL* mRNA levels; (C) *CPT-1b* mRNA levels; (D) *PGC-1α* mRNA levels in the *tibialis anterior* muscle; (E) Muscle lipid content (relative unit); and (F) Oil Red staining performed on frozen sections of the *tibialis anterior* muscles from the HF diet-treated group (HF), the HF- and HU-treated group (HF-HU) and the HF- and LPS-treated group (HF-LPS) compared with the levels in the control group (CT). All results are expressed as the means±SEM for 7–9 mice per group from the HF diet-treated group (HF), the HF- and HU-treated group (HF-HU) and the HF- and LPS-treated group (HF-LPS) compared with the levels in the control group (CT). Scale bar: 50 µm. The data with different superscripted letters are significantly different based on a one-way ANOVA followed by the Bonferroni *post hoc* test.

Cannabinoid agonist treated-mice that were challenged with an HF diet (HF-HU) exhibited a significant decrease of *GLUT-4*, *CPT-1b* and *PGC-1α* mRNA expression in the TA muscle compared with the CT group (Figures 7A, 7C and 7D). HF treatment alone decreased *GLUT-4* and *PGC-1α* (but did not reach significance) and decreased the *CPT-1b* expression level to the same extent as found in the HF-HU treatment group. Conversely, the *LPL* mRNA levels increased in the HF-HU group compared with the CT mice (Figure 7B). The LPS treatment did not affect these parameters compared with the HF and CT-treated mice (Figure 7A–D). HF-HU treatment increased the muscle lipid content (Figures 7E and 7F), as revealed by Oil Red O staining, compared with the CT-, HF- and HF-LPS-treated mice, whereas HF and HF-LPS treatment increased the muscle lipid content compared with the CT mice only.

## Discussion

### Rationale

Obesity is characterised by a massive expansion of the adipose tissue along with altered endocannabinoid-system tone, inflammation and metabolic endotoxaemia [6], [17]. Various studies have demonstrated that blocking the CB1 receptor improves glucose metabolism, and that eCB-system plays a central role in the onset of HF diet-induced insulin resistance [25], [36]–[40]. Moreover, we previously demonstrated that a CB1 receptor blockade reduces plasma glucose levels and low-grade inflammation by a mechanism that is dependent upon the gut barrier function (i.e., reduced gut permeability) and metabolic endotoxaemia in genetically obese *ob/ob* mice [6]. Because eCB-system stimulation and metabolic endotoxaemia are both hallmarks of diet-induced obesity [2], [6], we decided to investigate how CB receptor agonist treatment or LPS treatment contributes to the onset of the metabolic features that characterise diet-induced obesity (e.g., inflammation, insulin resistance and dyslipidaemia).The present study is the first to reveal that chronic cannabinoid receptor stimulation affects glucose and lipid metabolism via mechanisms associated with muscle lipids and macrophage accumulation.

### Validation of the treatment

We found that chronic CB receptor agonist treatment increased the mRNA levels of both target receptors (*CB1* and *CB2*) in the TA muscle, whereas PPARγ and GPR55 were not affected by the HU treatment (Figure S5). This increase primarily indicates that chronic treatment does not lead to a down-regulation of the receptor expression in the targeted organs at the end of the treatment. It is noteworthy that this down-regulation may have occurred at the beginning of the treatment, with a rebound increase during the subsequent weeks. However, several lines of evidence support that the CB receptor agonist was still effective at the end of 6 weeks of treatment. HU210 was analysed by HPLC-HRMS in the cerebellar tissue of the mice. The HPLC-MS^2^ trace clearly demonstrated the presence of HU210 in HU and HF-HU treated mice (Figure S2). Likewise, the HU210 treatment lowered the levels of AEA and 2-AG in the adipose tissue (Figure S6), suggesting that the endogenous eCBs are down-regulated and that the observed effects are attributable to HU210. Finally, to further demonstrate the stability of HU210 after the 6-week treatment, we injected the contents of the mini-pumps that were used for the experiment and harvested at the end of the treatment period in the wild-type mice. We found that acute HU210 treatment reduced the locomotor activity and the cataleptic state, demonstrating that HU210 was still active after the treatment (not shown). These results indicate that the CB receptor agonist was still effective while being delivered *in vivo* after six weeks of treatment.

### Effects of HU210 and LPS on glucose tolerance under basal conditions

In the present study, we found that under basal conditions (CT diet), chronic treatment with a CB receptor agonist induced glucose intolerance without affecting the insulin levels before (fasting) or after an oral glucose load, compared with the CT group. Insulin is a negative regulator of eCB levels. Activation of CB receptors in adipocytes, skeletal muscle or liver reduces insulin sensitivity. It has been demonstrated that an acute injection with AEA or a selective CB1 agonist, ACEA, in Wistar rats prior to a glucose tolerance test induces severe glucose intolerance [41]. Regulatory ability of insulin is absent during the obese state, [8], [42],Moreover, eCBs are known to modulate insulin release in the endocrine pancreas and appear to fulfil a paracrine regulatory function, although there are some discrepancies in the literature. The *in vitro* stimulation of CB receptors by HU210 in rat *β*-pancreatic cells and the stimulation of mouse *β*-cells with a CB1 agonist enhance insulin release [23], [43], whereas the *in vitro* stimulation of mouse *β*-cells with AEA, 2-AG, and CB1 or CB2 agonists inhibits insulin release [44]–[45]. During hyperglycaemia (i.e., type 2 diabetes and/or obesity) the eCB-system in pancreatic islets becomes dysregulated and may disrupt the regulatory loop between insulin and eCB [23] [39], [46]. The effects of chronic *in vivo* CB receptor stimulation on insulin release should be investigated and might partly contribute to the observed glucose intolerance, without affecting the glucose-induced insulin levels. In the present study, we could not rule out the possibility that chronic stimulation of CB receptors interferes with insulin signalling pathways (as previously proposed), thereby explaining the lack of effect that was observed on insulin secretion after chronic CB stimulation [42].

Interestingly, LPS treatment increases both fasting- and glucose-induced insulin levels by approximately 40%, suggesting a different mode of action for cannabinoids compared with LPS in glucose metabolism. These results confirm the effect of LPS on insulin sensitivity, as previously described by our group [2]. Notably, the CB receptor agonist-induced glucose intolerance was not related to weight gain or to the development of fat mass because these parameters were not affected by the treatment.

### Underlying mechanisms responsible for glucose intolerance?

#### Inflammation

Therefore, we investigated the underlying mechanisms responsible for this glucose intolerance. Interestingly, we found that both treatments induced an inflammatory state, which manifested as macrophage infiltration into the adipose tissue (Figure S3) and the skeletal muscle (Figure 3). CB receptor agonist treatment induced metabolic endotoxaemia, which confirmed our previous results demonstrating that HU210 administration increases gut permeability and plasma LPS levels [6].We have previously reported that metabolic endotoxaemia directly relies on the gut barrier integrity [6]. Moreover, we have also demonstrated that cannabinoid receptor agonists increase the gut permeability by CB1-dependent, but not CB2-dependent, mechanisms [6], an effect that has been recently confirmed [47].

Another key finding is that chronic CB receptor stimulation induces macrophage infiltration in the skeletal muscle. Although there is evidence that macrophage accumulation occurs in the skeletal muscle during HF diet-induced obesity, the mechanisms remain elusive [1], [48]. From our data (metabolic endotoxemia and muscle macrophage infiltration), we propose that cannabinoids are involved in the onset of metabolic inflammation. We cannot exclude the possibility that the recruited macrophages in the muscles are localised to the intermuscular adipose depots (which are known to accumulate in obesity) and could therefore contribute to insulin resistance through a paracrine mechanism [1]. At this point, we cannot certify whether these effects on muscle inflammation are CB1- or CB2-dependent (or mediated by other receptors, i.e., PPARγ and GPR55 [Figure S5]) because we found that the expression of both receptors was increased in the TA muscle. Thus, further investigations are required to unravel the role of CB receptors in muscle inflammation. We suggest that chronic CB receptor stimulation triggers macrophage infiltration into the skeletal muscle via a mechanism involving metabolic endotoxaemia and disturbed gut integrity; indeed, we have previously proposed the existence of a crosstalk between the eCB system and LPS, which starts in the intestine and regulates metabolic features (such as adipogenesis) that are associated with obesity [6].

#### Lipid metabolism

Subsequently, we analysed other metabolic markers in the muscles (*GLUT-4*, *LPL* and *CD36*) and found that under basal conditions, chronic CB receptor stimulation did not alter their expression (Figure S4). Plasma levels of NEFA contribute to the development of glucose intolerance by mediating substrate competition oxidation between glucose and lipids. In the present study, we measured a 30% increase in NEFA plasma levels in the HU-treated mice (Table 2). Despite this elevation in NEFA, we cannot speculate about a putative substrate competition between glucose and fatty acids. Moreover, neither *LPL* nor *CD36* were affected by the treatments (Figure S1). Altogether, these results do not support the role of altered lipid transport into the skeletal muscle. Various studies have previously shown that hepatic CB1 activation increases *de novo* lipogenesis and decreases fatty acid oxidation [49], [50]. We previously demonstrated that HU210 administration increases the mRNA expression of lipogenic genes in adipose tissue [6]. Here, we observed a decrease in the expression of *CPT-1b* and an increase in *PGC-1α* expression in the TA muscle, suggesting altered lipid oxidation after chronic CB receptor stimulation. This elevation of the *PGC-1α* expression level suggests a role for the eCB-system in regulating metabolism because *PGC-1α* is an established marker of mitochondria biogenesis as well as glucose and fatty acid metabolism [51]. It is noteworthy that the observed elevation of *PGC-1α* may not be explained based on other, previous studies [52], [53]. These discrepancies could be due to differences in the experimental procedures (chronic and persistent infusion *versus* chronic injections once or twice daily), but they also suggest that further investigations are required to analyse the effects of eCB on lipid metabolism. Interestingly, the LPS treatment did not affect the *CPT-1b* and *PGC-1α* mRNA levels, suggesting that low-dose LPS cannot directly affect mitochondrial lipid metabolism. Subsequently, we investigated the muscle lipid content by Oil Red O staining and found that none of the treatments affected the muscle lipid content (data not shown). Taken together, these data suggest that under basal conditions, chronic CB receptor stimulation could affect glucose and lipid metabolism by unresolved mechanisms, such as muscle inflammation.

### Challenging HU- or LPS-treated mice with a HF diet worsens glucose intolerance, muscle inflammation and lipid accumulation

We determined that challenging mice with an HF diet after 3 weeks of pre-treatment with a CB receptor agonist or with LPS did not significantly alter the body weight gain or fat mass (Table 2) compared with the HF diet alone. Mice treated with a HF diet concomitantly with HU210 chronic administration have increased CB1 and CB2 expression levels in the TA muscle, suggesting that these effects were attributable to the CB receptor agonist treatment (Figure S5). By contrast, the expression of GPR55 was increased by the HF treatment, with no additional effects of LPS or HU210, whereas the expression of PPARγ was not affected by the treatments (Figure S5Nonetheless, an important finding was that fat ingestion during chronic CB receptor stimulation exacerbated glucose intolerance and completely abrogated the glucose-induced insulin levels that were observed after the HF diet (Figure 5C), possibly explaining the worsened glucose intolerance that was observed. These results suggest that, under pathological conditions (HF diet), chronic CB receptor stimulation possibly affects endocrine pancreas insulin secretion; however, this hypothesis has yet to be investigated *in vivo*. Among the different mechanisms that were investigated, we found that the CD11c marker levels were increased under the HF diet and the CB receptor agonist treatment, suggesting that eCBs are potent enough to induce an inflammatory state in muscles that are in the basal condition; this phenomenon was found to be exacerbated under the HF diet. These findings indicate a novel role of the eCB-system in inducing inflammation at various levels (i.e., metabolic endotoxaemia and macrophage infiltration into the muscles). The effect on the lipid metabolism appeared to be more dependent on the HF diet than on chronic CB stimulation. Interestingly, the HF treatment combined with stimulation of the CB receptor increased (4.5-fold increase) the muscle lipid content compared with the CT mice as well as the HF- and HF-LPS-treated mice. We suggest that, under pathological conditions (HF diet), chronic CB receptor stimulation affects lipid metabolism by increasing lipid accumulation in the skeletal muscle, thereby affecting glucose tolerance. Indeed, the effects of endogenous ligands of the eCB-system appear to be greater in muscular cell derived from obese individuals than in similar cell lines from lean subjects [53], which is consistent with some of our observations. Finally, the concomitant LPS treatment along with an HF diet caused no additional effects on the lipid metabolism and the muscle lipid content compared with the HF diet alone, once again suggesting a different mechanism of action for LPS *versus* eCB on peripheral metabolism.

## Conclusion

We demonstrated for the first time that, during basal conditions, chronic CB receptor agonist treatment induced glucose intolerance, metabolic endotoxaemia, inflammation and altered lipid metabolism in skeletal muscles. More importantly, challenging mice with an HF diet after pre-treatment with a CB receptor agonist exacerbated glucose intolerance, stimulated inflammation and increased muscle lipid content, whereas pre-treatment with LPS affected the levels of inflammation and insulin secretion but not the glucose tolerance or the lipid metabolism. These results provide evidence that the effects of chronic stimulation of the CB receptor rely on the metabolic status of the mice. Taken together, our experiments demonstrate newly discovered roles played by the endocannabinoid system in both the onset and the physiopathology of obesity and type 2 diabetes.

## Supporting Information

Figure S1 **Schematic view of the study design.** This study was designed to analyse the effects of either a cannabinoid receptor agonist or LPS on glucose and lipid metabolism. To avoid interaction with dietary components (i.e. dietary lipids) and to decorticate whether these treatments induce metabolic alterations before the onset of obesity, mice were pre-treated with HU or LPS for 3 weeks before challenging mice with a HF diet. At treatment day 0, the mice were implanted subcutaneously with an mini-osmotic pump. After 3 weeks of being fed the CT diet, one-half of the mice were randomly assigned to 3 groups receiving a HF diet (HF) for an additional 3 weeks. The remaining mice received the treatments while on the CT diet.
(TIF)

Figure S2 **Validation of the HU210 stability and recovery in tissues after 6 weeks of administration.** To confirm the stability of the compound and its accurate *in vivo* delivery after 6 weeks of osmotic-mini-pump usage, the cerebella of four representative mice per group were analysed by HPLC-HRMS for the presence of HU210. Displayed is the HPLC-MS^2^ trace for HU210 in (A) control mice, (B) HU210 mice and (C) HU210 mice that were fed a high-fat diet. The HU210 peak at 11.7 min corresponds to the fragmentation 385.27482 367.26420, which indicates the loss of one of the two hydroxyl groups in HU210. The peak corresponding to the internal standard (CP-55940, m/z = 375.29047) is present in all the analysed tissues (not shown here). The HPLC-MS^2^ trace clearly demonstrated the presence of HU210 in HU and HF-HU treated mice.
(TIF)

Figure S3 **F4/80 mRNA expression in the subcutaneous adipose tissue.** *F4/80* mRNA levels in the subcutaneous adipose tissue. All results are expressed as the means±SEM for 7–9 mice per group from the HU-treated group (HU) and the LPS-treated group (LPS) compared with the levels in the control group (CT). The data with different superscripted letters are significantly different based on a one-way ANOVA followed by the Bonferroni *post hoc* test.
(TIF)

Figure S4 **GLUT-4, LPL and CD36 mRNA levels after chronic CB receptor agonist treatment or LPS treatment.** (A) *GLUT-4* mRNA levels; (B) *LPL* mRNA levels; and (C) *CD36* mRNA levels in the *tibialis anterior* muscle. All results are expressed as the means±SEM for 7–9 mice per group from the HU-treated group (HU) and the LPS-treated group (LPS) compared with the levels in the control group (CT). The data with different superscripted letters are significantly different based on a one-way ANOVA followed by the Bonferroni *post hoc* test.
(TIF)

Figure S5 **Cannabinoid receptor mRNA levels.** (A) *PPARγ* mRNA levels; (B) *GPR55* mRNA levels in the *tibialis anterior* muscle. All the results are expressed as the means±SEM for 7–9 mice per group from the HU-treated group (HU) and the LPS-treated group (LPS) compared with the levels in the control group (CT). (C) *PPARγ* mRNA levels; (D) *GPR55* mRNA levels; (E) *CB1* mRNA levels; and (F) *CB2* mRNA levels in the *tibialis anterior* muscles from the HF diet-treated group (HF), the HF- and HU-treated group (HF-HU) and the HF- and LPS-treated group (HF-LPS) compared with the levels in the control group (CT). The data with different superscripted letters are significantly different based on a one-way ANOVA followed by the Bonferroni *post hoc* test.
(TIF)

Figure S6 **AEA and 2-AG levels in the cerebellum and the subcutaneous adipose tissue.** (A) AEA levels and (B) 2-AG levels in the cerebella (CVL) and the subcutaneous adipose tissues (SATs) of the HU-treated group (HU) and the HF-and HU-treated group (HF-HU) compared with the levels in the control group (CT). All the results are expressed as the means±SEM for 5 mice per group. The data with different superscripted letters are significantly different based on a one-way ANOVA followed by the Bonferroni *post hoc* test.
(TIF)
