# Supplementary figures and images for: Chronic Endocannabinoid System Stimulation Induces Muscle Macrophage and Lipid Accumulation in Type 2 Diabetic Mice Independently of Metabolic Endotoxaemia
Source: PLoS One. 2013 Feb 5;8(2):e55963. doi: 10.1371/journal.pone.0055963 (PMC3564911; doi:10.1371/journal.pone.0055963)

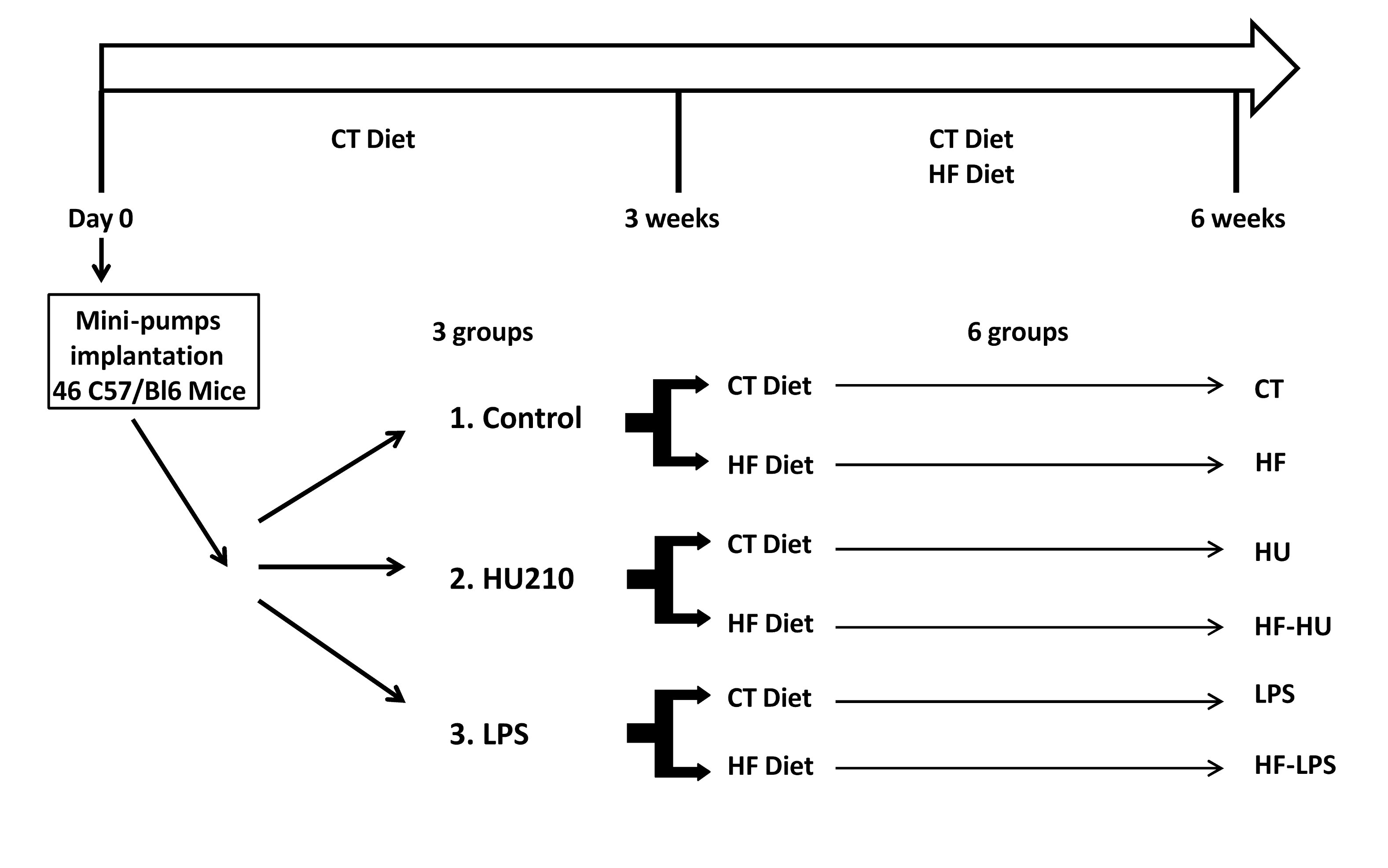

Supplement: Figure S1 — Schematic view of the study design. This study was designed to analyse the effects of either a cannabinoid receptor agonist or LPS on glucose and lipid metabolism. To avoid interaction with dietary components (i.e. dietary lipids) and to decorticate whether these treatments induce metabolic alterations before the onset of obesity, mice were pre-treated with HU or LPS for 3 weeks before challenging mice with a HF diet. At treatment day 0, the mice were implanted subcutaneously with an mini-osmotic pump. After 3 weeks of being fed the CT diet, one-half of the mice were randomly assigned to 3 groups receiving a HF diet (HF) for an additional 3 weeks. The remaining mice received the treatments while on the CT diet. (TIF) [file pone.0055963.s001.tif]

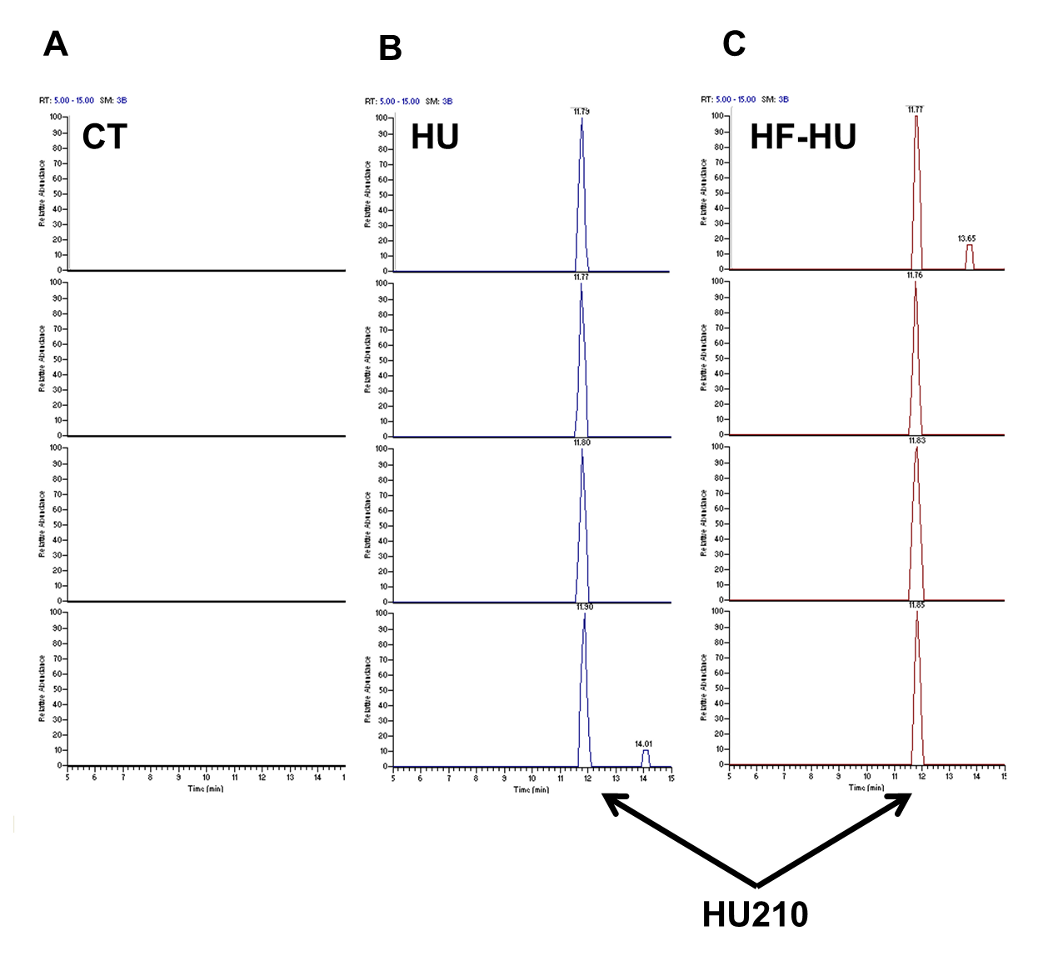

Supplement: Figure S2 — Validation of the HU210 stability and recovery in tissues after 6 weeks of administration. To confirm the stability of the compound and its accurate in vivo delivery after 6 weeks of osmotic-mini-pump usage, the cerebella of four representative mice per group were analysed by HPLC-HRMS for the presence of HU210. Displayed is the HPLC-MS2 trace for HU210 in (A) control mice, (B) HU210 mice and (C) HU210 mice that were fed a high-fat diet. The HU210 peak at 11.7 min corresponds to the fragmentation 385.27482 367.26420, which indicates the loss of one of the two hydroxyl groups in HU210. The peak corresponding to the internal standard (CP-55940, m/z = 375.29047) is present in all the analysed tissues (not shown here). The HPLC-MS2 trace clearly demonstrated the presence of HU210 in HU and HF-HU treated mice. (TIF) [file pone.0055963.s002.tif]

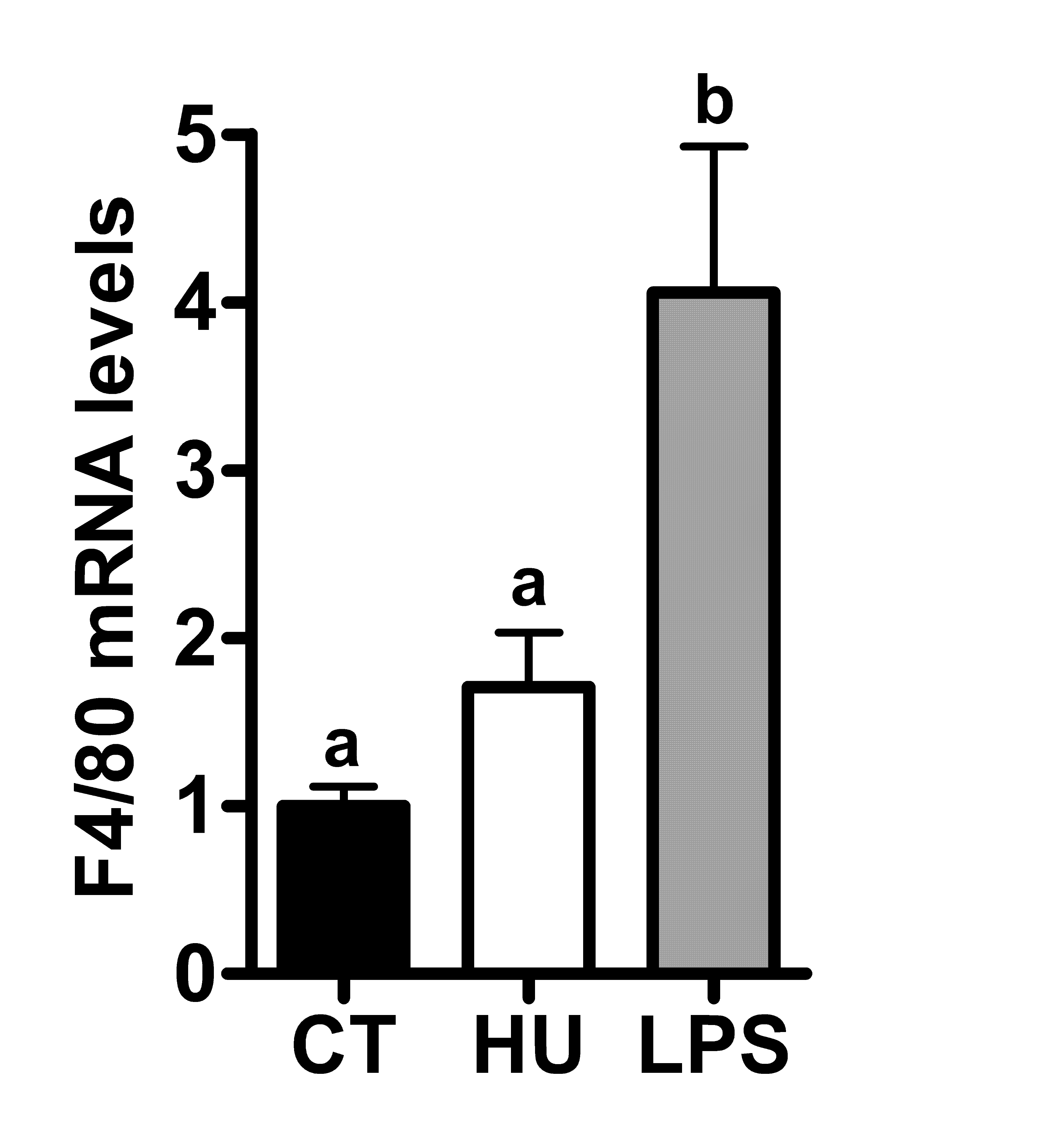

Supplement: Figure S3 — F4/80 mRNA expression in the subcutaneous adipose tissue. F4/80 mRNA levels in the subcutaneous adipose tissue. All results are expressed as the means±SEM for 7–9 mice per group from the HU-treated group (HU) and the LPS-treated group (LPS) compared with the levels in the control group (CT). The data with different superscripted letters are significantly different based on a one-way ANOVA followed by the Bonferroni post hoc test. (TIF) [file pone.0055963.s003.tif]

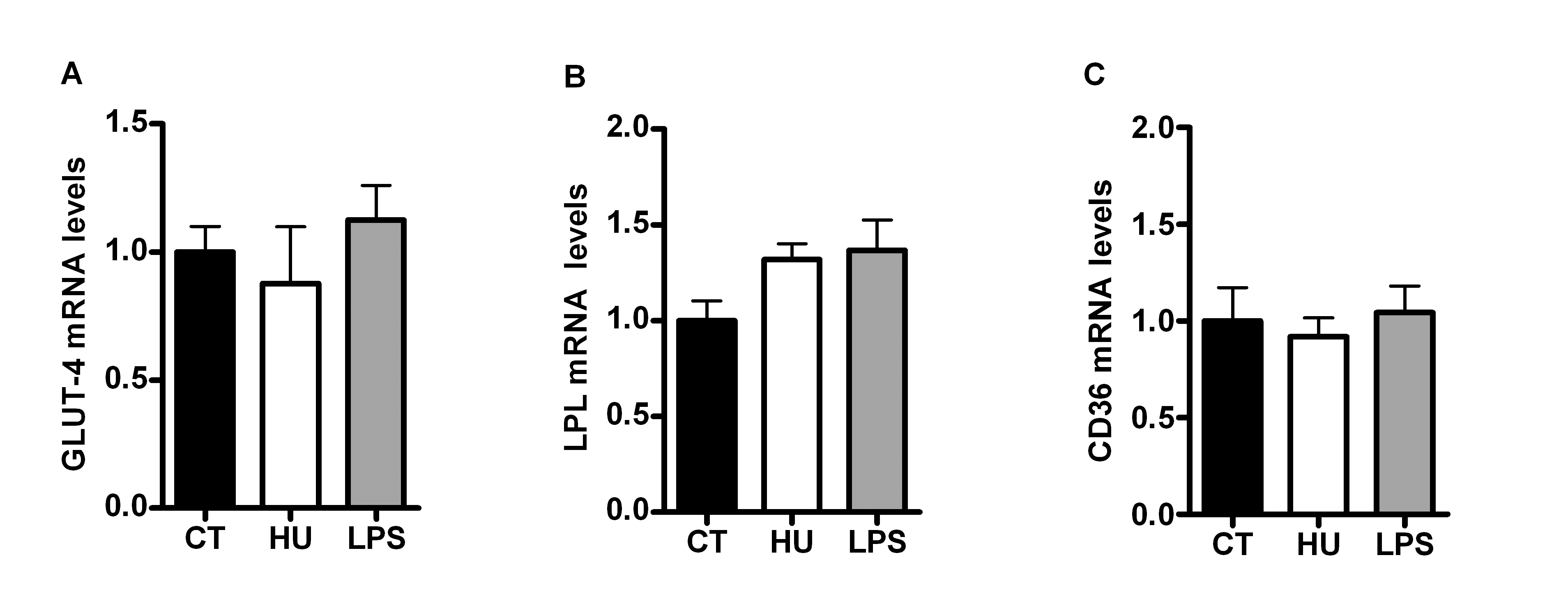

Supplement: Figure S4 — GLUT-4, LPL and CD36 mRNA levels after chronic CB receptor agonist treatment or LPS treatment. (A) GLUT-4 mRNA levels; (B) LPL mRNA levels; and (C) CD36 mRNA levels in the tibialis anterior muscle. All results are expressed as the means±SEM for 7–9 mice per group from the HU-treated group (HU) and the LPS-treated group (LPS) compared with the levels in the control group (CT). The data with different superscripted letters are significantly different based on a one-way ANOVA followed by the Bonferroni post hoc test. (TIF) [file pone.0055963.s004.tif]

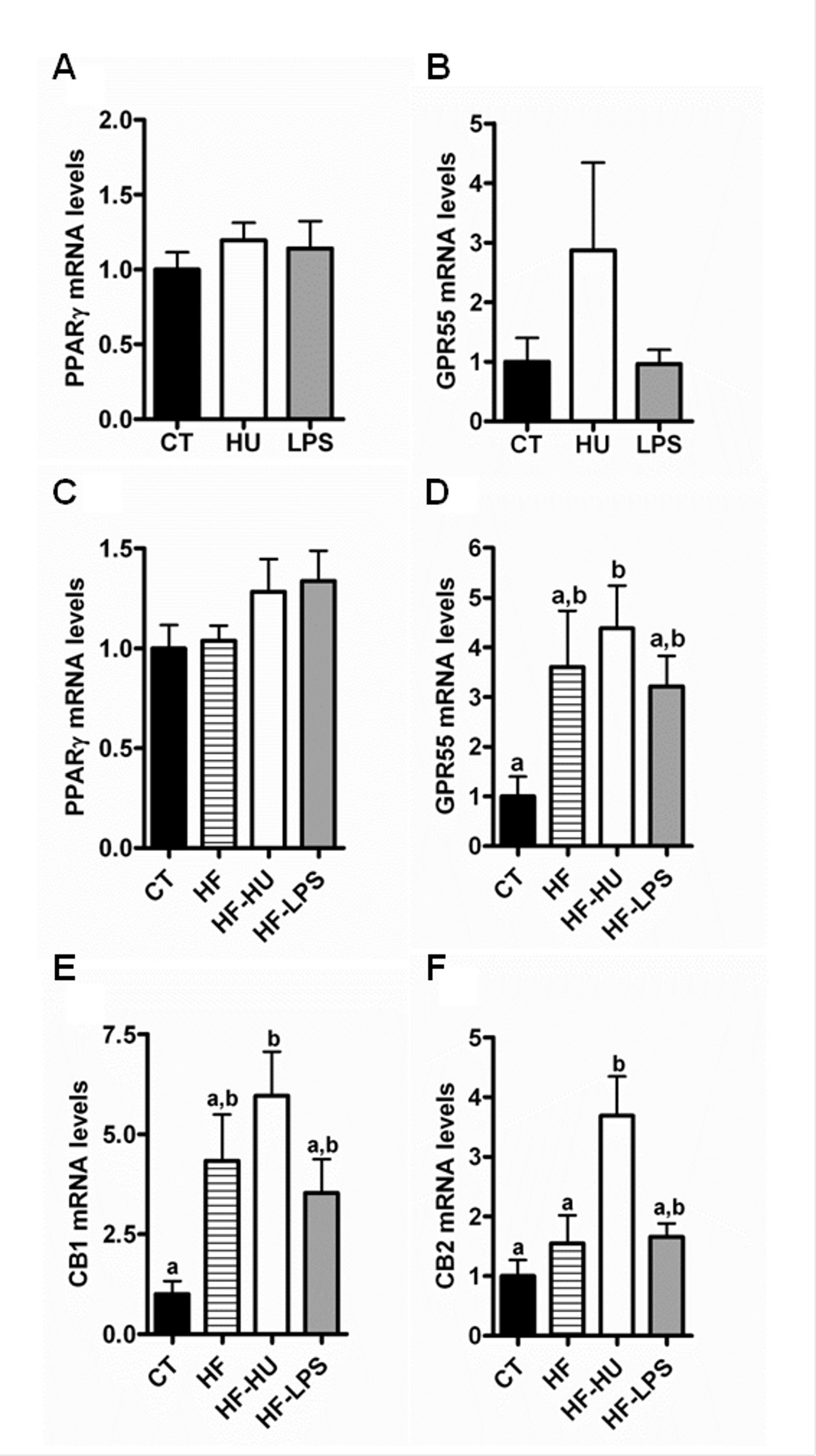

Supplement: Figure S5 — Cannabinoid receptor mRNA levels. (A) PPARγ mRNA levels; (B) GPR55 mRNA levels in the tibialis anterior muscle. All the results are expressed as the means±SEM for 7–9 mice per group from the HU-treated group (HU) and the LPS-treated group (LPS) compared with the levels in the control group (CT). (C) PPARγ mRNA levels; (D) GPR55 mRNA levels; (E) CB1 mRNA levels; and (F) CB2 mRNA levels in the tibialis anterior muscles from the HF diet-treated group (HF), the HF- and HU-treated group (HF-HU) and the HF- and LPS-treated group (HF-LPS) compared with the levels in the control group (CT). The data with different superscripted letters are significantly different based on a one-way ANOVA followed by the Bonferroni post hoc test. (TIF) [file pone.0055963.s005.tif]

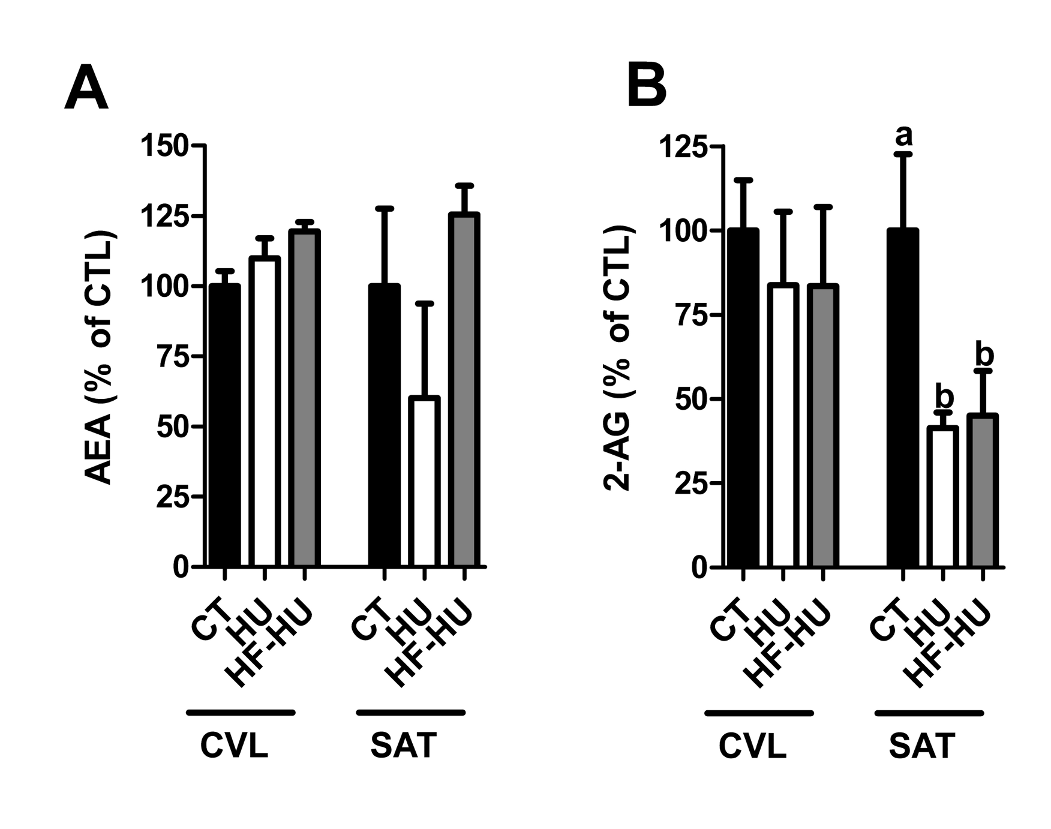

Supplement: Figure S6 — AEA and 2-AG levels in the cerebellum and the subcutaneous adipose tissue. (A) AEA levels and (B) 2-AG levels in the cerebella (CVL) and the subcutaneous adipose tissues (SATs) of the HU-treated group (HU) and the HF-and HU-treated group (HF-HU) compared with the levels in the control group (CT). All the results are expressed as the means±SEM for 5 mice per group. The data with different superscripted letters are significantly different based on a one-way ANOVA followed by the Bonferroni post hoc test. (TIF) [file pone.0055963.s006.tif]
